# Applying an intersectionality lens to the theoretical domains framework: a tool for thinking about how intersecting social identities and structures of power influence behaviour

**DOI:** 10.1186/s12874-020-01056-1

**Published:** 2020-06-26

**Authors:** Cole Etherington, Isabel Braganca Rodrigues, Lora Giangregorio, Ian D. Graham, Alison M. Hoens, Danielle Kasperavicius, Christine Kelly, Julia E. Moore, Matteo Ponzano, Justin Presseau, Kathryn M. Sibley, Sharon Straus

**Affiliations:** 1 Clinical Epidemiology Program, Ottawa Hospital Research Institute, 501 Smyth Road, Rm L1287, Ottawa, ON K1H 8L6 Canada; 2 Department of Kinesiology, University of Waterloo, Waterloo, Canada; 3 Schlegel-UW Research Institute for Aging and KITE Toronto Rehab-University Health Network, Toronto, Canada; 4 School of Epidemiology and Public Health, University of Ottawa, Ottawa, Canada; 5 Department of Physical Therapy, University of British Columbia, Vancouver, Canada; 6 Centre of Health Evaluation and Outcome Sciences, University of British Columbia, Vancouver, Canada; 7 Arthritis Research Canada, Richmond, Canada; 8 Li Ka Shing Knowledge Institute, St. Michael’s Hospital, Toronto, Canada; 9 Department of Community Health Sciences, University of Manitoba, Winnipeg, Canada; 10 The Center for Implementation, Toronto, Canada; 11 Centre for Healthcare Innovation, Winnipeg, Manitoba Canada; 12 Department of Medicine, University of Toronto, Toronto, Canada

**Keywords:** Intersectionality, Knowledge translation, Theoretical domains framework, Behaviour change, Implementation science, Determinant framework, Intervention development, Barriers, Facilitators

## Abstract

**Background:**

A key component of the implementation process is identifying potential barriers and facilitators that need to be addressed. The Theoretical Domains Framework (TDF) is one of the most commonly used frameworks for this purpose. When applying the TDF, it is critical to understand the context in which behaviours occur. Intersectionality, which accounts for the interface between social identity factors (e.g. age, gender) and structures of power (e.g. ageism, sexism), offers a novel approach to understanding how context shapes individual decision-making and behaviour. We aimed to develop a tool to be used alongside applications of the TDF to incorporate an intersectionality lens when identifying implementation barriers and enablers.

**Methods:**

An interdisciplinary Framework Committee (*n* = 17) prioritized the TDF as one of three models, theories, and frameworks (MTFs) to enhance with an intersectional lens through a modified Delphi approach. In collaboration with the wider Framework Committee, a subgroup considered all 14 TDF domains and iteratively developed recommendations for incorporating intersectionality considerations within the TDF and its domains. An iterative approach aimed at building consensus was used to finalize recommendations.

**Results:**

Consensus on how to apply an intersectionality lens to the TDF was achieved after 12 rounds of revision. Two overarching considerations for using the intersectionality alongside the TDF were developed by the group as well as two to four prompts for each TDF domain to guide interview topic guides. Considerations and prompts were designed to assist users to reflect on how individual identities and structures of power may play a role in barriers and facilitators to behaviour change and subsequent intervention implementation.

**Conclusions:**

Through an expert-consensus approach, we developed a tool for applying an intersectionality lens alongside the TDF. Considering the role of intersecting social factors when identifying barriers and facilitators to implementing research evidence may result in more targeted and effective interventions that better reflect the realities of those involved.

## Background

Knowledge translation (KT) is “a dynamic and iterative process” involving the synthesis, dissemination, exchange, and application of knowledge in order to improve health services, the healthcare system, and population health” [1]. We use the term KT to broadly refer to the dissemination and implementation of research-based evidence, though we recognize there are many terms that can be used to describe this process [2]. As the science and practice of KT has evolved, there has been increasing emphasis on using models, theories, and frameworks (MTFs) to guide and evaluate implementation processes and to understand implementation outcomes [3, 4].

When applying any MTF in KT, it is critical to understand the context in which behaviours occur [5–7]. Recently, KT researchers have called for greater incorporation of social and structural factors to enhance our understanding of the contextual influences on behaviour [2, 8–12]. For example, sex and gender have been cited as key factors to consider in KT research and practice [2]. Sex and gender, while important, are just two of the many different social categories which individuals concurrently occupy (e.g., ethnicity, geography, age, class). These intersecting social categories also interact with systems and structures of power (e.g., sexism, racism, ableism, ageism) [13–16]. The interface between social identity factors and structures of power is referred to as ‘intersectionality’ [13, 14].

Though many approaches emphasize the need to consider a variety of social categories when studying health issues [17–19], intersectionality has been repeatedly identified as an important theoretical framework for health research [20–23]. As a central theoretical concept and social justice framework, intersectionality provides a way to consider individual experiences within larger social contexts, highlighting how various intersections structure our everyday lives and interactions [24–26]. Initially developed by black feminist and critical race scholars in the 1980s [13], intersectionality has since grown to more broadly emphasize “the multiple ‘axes’ of power and difference that shape individuals’ positionalities” [27]. In other words, an individual’s lived experience cannot be reduced to a single characteristic, experiences can change over time and in different contexts, and privilege (i.e., social advantage) and oppression (i.e., social disadvantage) can be experienced simultaneously [13–16].

Though it has yet to be considered within KT, intersectionality offers a nuanced and comprehensive account of context, and uniquely and importantly can be used to consider how these factors *intersect* to shape individual decision-making and behaviour. Accounting for the diverse intersections of individuals’ lived experiences has the potential to increase the effectiveness and generalizability of interventions and enhance their sociological fidelity [28, 29].

Incorporating intersectionality within foundational KT MTFs is also consistent with established recommendations for adopting theory-driven approaches to KT [3, 4]. We aimed to develop tools for incorporating an intersectionality lens when using KT MTFs to develop and implement interventions. In this paper, we illustrate this process using the Theoretical Domains Framework (TDF) [30, 31], one of the most commonly used frameworks for assessing barriers and facilitators as part of the Knowledge to Action (KTA) cycle [32].

## Methods

**Fig. 1:**
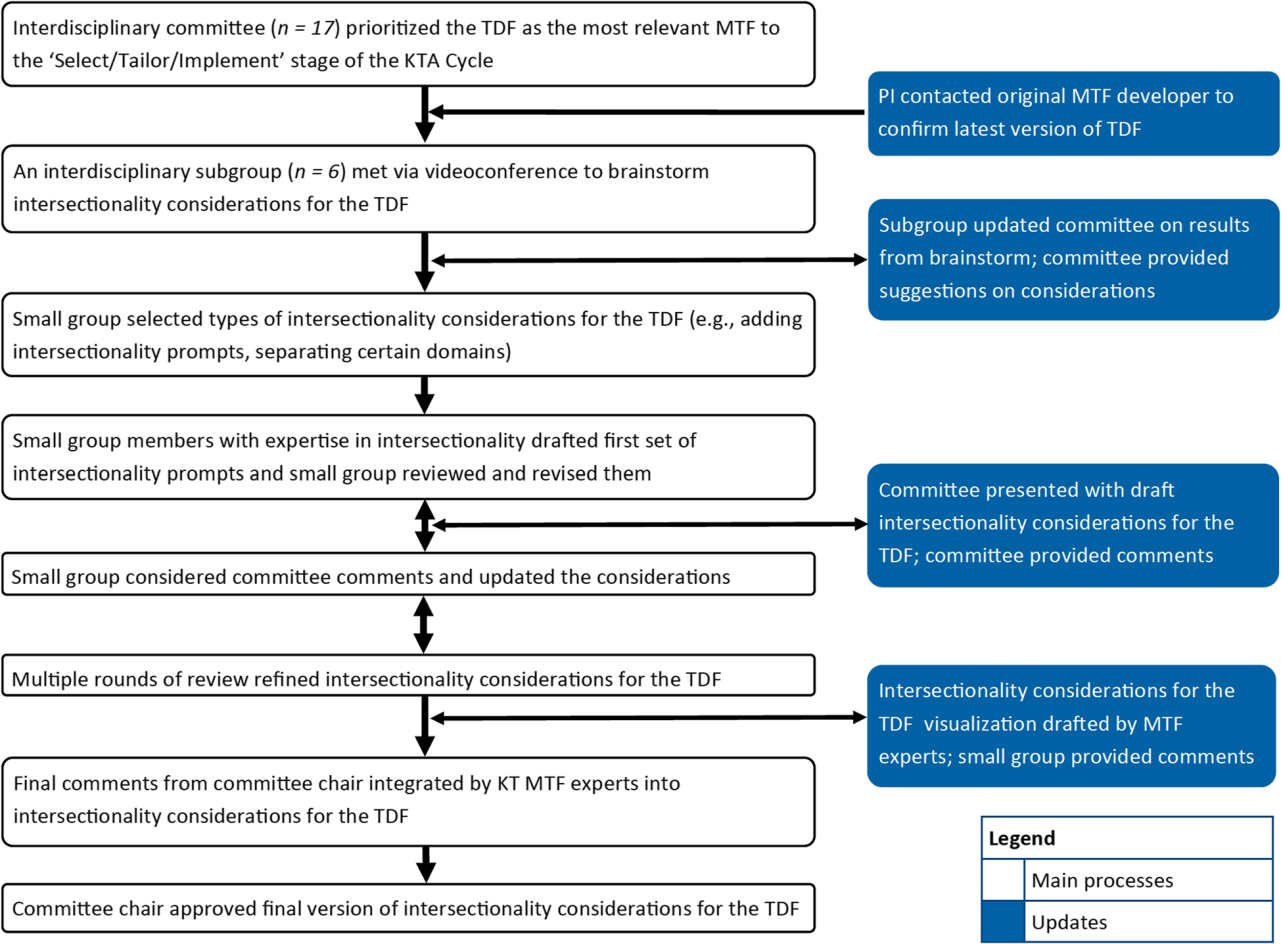
Process summary taken by Framework Committee to develop intersectionality considerations for the TDF

Our methodological approach is summarized in Fig. 1 and described below. While there are various and evolving definitions of intersectionality, the project team elected to focus on how intersecting categories (e.g. age, gender) interact to form a person’s identity. Experiences of these intersecting identities reflect larger systems of oppression/privilege (e.g. sexism, ageism) [16]. The concept of “intersecting categories” was also selected for feasibility reasons and its alignment with the Cochrane Equity Method’s PROGRESS-Plus factors [8, 18].

### Selection of the framework

An interdisciplinary Framework Committee was established to select MTFs to enhance with an intersectional lens. The committee was comprised of 17 members with expertise in KT or intersectionality and disciplinary backgrounds in community health, kinesiology, medicine, physical therapy, psychology, and sociology.

First, through a consensus-building activity involving the Framework Committee and community stakeholders, three key steps of the Knowledge to Action (KTA) Framework [32] were prioritized: a) *identify problem (know-do gap)*, b) *assessing barriers and facilitators to knowledge use* and c) *select, tailor and implement interventions* [20]. Second, KT MTFs were mapped to these three steps and criteria for prioritizing MTFs for each step were developed by the Framework Committee. A modified Delphi approach [33] involving two rounds was then completed through online surveys, and a final majority vote was conducted to determine agreement on the top selected MTF for each step.

The Framework Committee prioritized the TDF to complement the the Behaviour Change Wheel (BCW), along with two other MTFs (Consolidated Framework for Implementation Research [34] and Iowa Model of Evidence-Based Practice to Promote Quality Care [35]). The Framework Committee was divided into three subgroups, one for each selected MTF. A full description of the MTF selection process along with additional tools for the Consolidated Framework for Implementation Research and Iowa Model of Evidence-Based Practice to Promote Quality Care can be found on our website [36]. Details regarding the MTF selection process are described elsewhere [37]. In brief, an online rating survey was used to discuss potential prioritization criteria, informed by Birken et al.’s T-CaST tool [38]. Ultimately, the group used a majority vote to select three criteria: acceptability (i.e. the MTF is likely to be familiar to KT intervention developers); applicability (i.e. the MTF can likely be generalized by KT intervention developers to different populations, settings and disciplines as needed); and usability (i.e. KT interventions developers are likely to be able to understand and operationalize the MTF for the KTA stage under consideration). The experience of KT trainees and intervention developers on the Committee informed the group’s prioritization. The TDF was concluded to be widely used by practitioners, generalizable to multiple practice changes and settings, and easy to understand.

The TDF synthesizes 33 theories of behaviour and behaviour change clustered into 14 domains [30, 31], and has been used across a wide range of healthcare settings to identify determinants of behaviour and facilitate intervention design [5]. The TDF/BCW subgroup elected to consider the TDF separately from the Capability, Opportunity, Motivation and Behaviour components of the BCW based on its relevance to the select/tailor/implement stage of the KTA Framework. In other words, the Framework Committee chose to focus on the use of the TDF to identify barriers and enablers to a desired behaviour rather than on its role in facilitating the selecting of implementation interventions.

### Enhancing the TDF framework

The Principal Investigator (SS) approached the developer of the original TDF [31] to obtain support for enhancing the framework and to confirm that the committee was referring to the most recent version of the TDF. An interdisciplinary subgroup (six members) met via videoconference to identify types of intersectionality considerations that could be used alongside the TDF in general and then for each individual TDF domain. This approach was taken given application of the TDF to KT typically involves semi-structured interviews with particular questions and prompts to elicit perspectives about each of the 14 domains related to behaviour [5]. Discussions were facilitated by the Framework Committee Chair (JP) and the study research coordinator (DK). The TDF subgroup updated the Framework Committee on the results of their initial meeting, which provided suggestions on the intersectionality considerations.

The TDF subgroup selected from among these intersectionality considerations and members with expertise in intersectionality drafted the first set of overarching intersectional considerations designed to trigger reflection on intersectionality-related issues for users of the TDF. The group also drafted specific prompts for each TDF domain that could be incorporated into interview topic guides.

The Framework Committee reviewed the overarching considerations and list of prompts and provided comments. The subgroup considered these comments and conducted multiple rounds of review via web-meetings and email exchanges. When consensus on the items was achieved within the TDF subgroup, the Framework Committee drafted visualizations of the intersectionality considerations and the subgroup provided feedback. Draft visualization and prompts were created by the Framework Committee and final comments from the Framework Committee Chair were integrated. The Framework Committee Chair approved the final version of the intersectionality considerations for the TDF and associated visualization. The final version was incorporated into a tool for using intersectionality with KT frameworks. The primary target of the tool is KT practitioners.

## Results

Consensus on the intersectionality enhanced TDF was achieved after twelve rounds of revision (five full Framework Committee meetings, two TDF subgroup meetings, four review rounds (by email) for TDF subgroup, and one final review by Framework Committee Chair).

### Suggested adaptation for “social/professional role” and “identity”

The TDF contains the domain “social/professional role and identity”. Identity is a core concept within intersectionality which has traditionally been under-examined in applications of the TDF. Identity plays a key role in decisions and behaviours but can often be subsumed under “role”. To emphasize the importance of social identity and improve clarity when using an intersectionality lens, we present two sets of prompts for the domain “social/professional role and identity”. In other words, we have identified specific prompts related to “identity” and prompts related to “social/professional role”.

### Overarching intersectionality considerations when applied to the TDF

Four overarching considerations for using an intersectionality lens with the TDF were developed. Table 1 provides the text that the subgroup developed when considering factors to enhance the TDF with an intersectional lens, along with examples. This text was then included in the final version of the intersectionality tool. The full toolkit for using intersectionality when designing KT interventions is available on our website [36].

**Table 1:** Overarching considerations when using the intersectionality-enhanced TDF

**Consideration**	**Example**
Though many TDF domains focus on the individual, individuals are impacted by systems and structures of power.	Self-efficacy (i.e. one’s belief in their capability to exercise control over one’s own behaviour; *beliefs about capabilities TDF domain*) is associated with better health outcomes [39]. Self-efficacy varies across social identity categories (e.g. Black women have lower levels of self-efficacy than black men, Caucasian women and Caucasian men) [39]. This may be a reflection of power structures in society related to both race and gender. Thus, while self-efficacy is often viewed as a psychological factor, there are social structural factors that can influence individuals’ perceived capabilities. Interventions to enhance self-efficacy may need to consider how some groups have been historically marginalized and disempowered and that their position in society may influence whether they feel they can take action to prevent or control their own health conditions.
Reflect on how the TDF domains intersect with each other.	An individual’s intersecting *social identity* categories and *professional role* may be related to their experience of *social influences*. For example, a racialized Personal Support Worker may feel unable to speak up if they disagree with a Caucasian team member who is also a Registered Nurse.
Do those developing/delivering the intervention/policy reflect the diversity of those who will be impacted by it?	Reflect on whether everyone who could be on the team has been asked if and how they would like to be involved. Think about how different perspectives that represent a range of intersecting categories have been examined. Consider whether you team reflects the makeup of the patient, community, and health care providers that experiences the project topic.
Have those impacted by the intervention/policy been involved in its development?	Consider the patient, healthcare provider, and community population affected by the project topic area. Develop a plan to get them involved. Include multiple individuals to represent a particular group (e.g. five patient partners instead of one).

Through the consensus process described previously, 53 prompts were developed to guide TDF interviews or questionnaires, with a median of 3 (IQR 2–4) considerations or questions per theoretical domain (Table 2).

**Table 2:** Intersectionality suggested prompts for each TDF domain

**Theoretical Domain**	**Definition of domain**^**a**^	**Intersectionality Prompts**
Identity	One’s self concept, including one’s perception of relevant intersecting and interacting social categories.	Tell me a little bit about who you are as a person. What categories (e.g., race, gender) are important for someone else to know when they are exploring enhancing the way that you work? How do you think these factors affect you doing [target behavior]? Prompt: some people talk about their language/accent, gender, where they live, who they know, etc. [list categories described by respondent] How do these categories intersect to define you? Do you feel these categories influence others’ perceptions of you? If yes, how does this shape how you engage with [target behavior]? Are there any categories (e.g., gender) that you feel influence [target behaviour]? How do you think they intersect to influence [target behaviour] for you? Are there social identity categories that you have observed as important for influencing others’ engaging in [target behavior]?
Social/Professional Role	A coherent set or expectation of behaviours and displayed personal qualities of an individual in a social or work setting	Do you believe there are intersecting categories that influence your social or professional role? Do you think they influence in a positive, neutral, or negative way? How do you think your intersecting categories influence your role? How do you think your intersecting categories influence your sense of belonging with your team at work? Do you think your intersecting categories influence their beliefs on whether you should or should not perform [target behaviour]?
Emotion	A complex reaction pattern, involving experiential, behavioural, and physiological elements, by which the individual attempts to deal with a personally significant matter or event	How do you think the intersection of [categories listed by participant] (e.g., intersection of occupation and ethnicity) relates to the feelings you have toward [target behaviour]?
Reinforcement	Increasing the probability of a response by arranging a dependent relationship, or contingency, between the response and a given stimulus	Are there rewards for engaging in [target behaviour] that are relevant to the groups you belong to/identify with (e.g., financial awards for female junior scientists)? Are these rewards important to you? Are there incentives not to do [target behaviour] that relate to the groups you belong to or identify with (e.g., engaging in behaviour will reinforce negative gender stereotypes about leadership)?
Knowledge	An awareness of the existence of something	Do you think there is enough evidence for [target behaviour]? How might the intersection of [categories listed by participant] (e.g., intersection of education, age, socioeconomic status) influence whether you think there is enough evidence or not? Where and how did you learn about [target behaviour]? How might the intersection of [categories listed by participant] (e.g., intersection of ethnicity and religion) impact your knowledge about [target behaviour]? From your perspective, what knowledge is required to change or improve [target behaviour]?
Skills	An ability or proficiency acquired through practice	What, if anything, about the intersection of the categories you belong to or identify with makes it easy or hard to [target behaviour]? How have your life experiences shaped the social skills required to engage in [target behaviour]? How might structures of power (e.g., racism) impact your access to acquiring skills required for [target behaviour]? Do you think your intersecting categories make it harder or easier to physically do [target behaviour] compared to other people? Why? Have you attended or engaged in any training to do [target behaviour]? If not, why not? In what ways might your intersecting categories influenced whether you attended or how you experienced training related to [target behaviour]? Are there considerations for future training you feel are important based on your experience?
Memory, Attention, Decision Processes	The ability to retain information, focus selectively on aspects of the environment and choose between two or more alternatives	When was a time you forgot to do [target behaviour]? Are there any pieces about your life or personal story related to your intersecting categories that played a role? When was a time you actively decided to do or not to do [target behaviour]? Are there any pieces about your life or personal story that played a role in the decision to do or to not do [target behaviour]? If so, what are they? How and why did they influence your decision?
Behavioral Regulation	Anything aimed at managing or changing objectively observed or measured actions	Are there any specific traditions, practices, or resources from your socio-cultural background that do or would help you make [target behaviour] a habit?
Social Influences	Those interpersonal processes that can cause individuals to change their thoughts, feelings, or behaviours	How do the social groups you belong to/identify with influence [target behaviour]? Do you think the intersecting categories of others influence their beliefs related to [target behaviour]? How? What do you think are other peoples’ perceptions of you doing [target behaviour]? Do you think they think it is important to do or not to? Do you feel pressure by the social groups you belong to/identify with to do or not do [target behaviour]? How might these feelings or pressure intersect? What are others’ expectations about [target behaviour]? How do their expectations intersect with your expectations about [target behaviour]? How might others’ intersecting categories influence their expectations about your engagement in [target behaviour]? Do particular social groups of other people influence your expectations of yourself related to [target behaviour]? Do you feel you have power within the social groups you belong/to identify with? How may this feeling of power or lack of power influence [target behaviour]? How might internalized oppression (e.g., internalized racism) impact [target behaviour]? Are there social groups that you do not belong to/identify with that may influence [target behaviour]? How do the people in your life talk about [target behaviour]? What intersectional categories do they belong to? What do they say about [target behaviour]?
Environmental Context and Resources	Any circumstance of a person’s situation or environment that discourages or encourages the development of skills and abilities, independence, social competence, and adaptive behaviour	How do your intersecting categories influence your access to the resources you need to do [target behaviour]? Have the groups you belong to/identify with experienced specific benefits or challenges in your current context? How might these benefits or challenges intersect and influence [target behaviour]? *(*e.g.*, Have you faced racism, ableism, or structures operating in society that create inequalities and reinforce exclusion)* Have you experienced benefits based on the groups you belong to/identify with (e.g., others identify your professional role based on your gender)? How does where you live and work impact your experience of [target behaviour]? How does your level of education impact your experience of [target behaviour]?
Optimism	The expectation, hope or confidence that things will happen for the best or that desired goals will be attained	How does who you are as a person (e.g., intersection of gender and age) make you hopeful about doing [target behaviour]? How does the intersection of [categories listed by participant] (e.g., intersection of education and socioeconomic status) make you pessimistic about doing [target behaviour]?
Beliefs about Consequences	Acceptance of the truth, reality, or validity about outcomes of a behaviour in a given situation	What do you think the impact is of doing [target behaviour]? What, if any, of your intersecting categories do you think influences your belief that doing [target behaviour] will [outcome stated by participant, e.g. improve healing]? Why or in what ways? If you haven’t engaged in [target behaviour], can you describe what you think would happen if you did [target behaviour]? How did you come to this description? How might your intersecting categories influence this description?
Beliefs about Capabilities	Acceptance of the truth, reality, or validity about an ability, talent, or facility that a person can put to constructive use	What about who you are as a person (e.g., intersection of education and gender) makes it easy or difficult for you to engage in [target behaviour]? What about who you are as a person (e.g., intersection of your home in the community and age) makes you more or less confident to make this change? Why? How might experiences of discrimination or oppression based on intersecting categories impact beliefs about your capabilities to do [target behaviour], either for yourself or for others?
Intentions	A conscious decision to perform a behaviour or a resolve to act in a certain way	How motivated are you to do [target behaviour]? What about who you are as a person (e.g., intersection of education and age) makes you motivated or not motivated? How does who you are as a person (e.g. intersection of gender and age) influence whether you have a plan to do [target behaviour]?
Goals	Mental representations of outcomes or end states that an individual wants to achieve	How much of a priority is engaging in [target behaviour] for you? What about who you are as a person (e.g., intersection of socioeconomic status and gender) influences whether or not you want to engage in [target behaviour] relative to your other priorities?

^a^Definitions adapted from Atkins et al. [8]

### Case example: mobilization of vulnerable elders (MOVE)

In the late 2000s, the Division of Geriatric Medicine at the University of Toronto, along with collaborators, reviewed evidence relating to successful aging [40]. The team noted that keeping older adults physically active while in hospital improved older adults’ functional status after they left the hospital [40]. After reviewing administrative data, the Geriatric Medicine team found that many elderly patients admitted to acute care hospitals in Ontario were confined to their beds or chairs while in the hospital [40]. Accordingly, the Geriatric Medicine team identified the problem of not keeping older adults physically active while in hospital [40]. The Geriatric Medicine team, along with staff at four Ontario hospitals, formed a KT intervention development team to address this problem in different units across four hospitals.

At each of the four hospitals, the KT intervention development team investigated the barriers and facilitators to greater mobility of hospitalized older patients different hospital units. The KT intervention development team used surveys and interviews with relevant individuals, including nurses on the different hospital units. *When conducting surveys and interviews to identify barriers and facilitators, the intersectionality considerations and prompts described above can be used.*

After conducting the barriers assessment, the team found that the largest barrier to nurse assessing patients’ mobility within 24 h of admission was their belief that if older patients were mobilized, they would be more likely to fall. The nurses did not want to cause harm and wanted to adhere to the hospital’s falls prevention policies. Knowing this barrier, the KT intervention development team selected and tailored a KT intervention to target nurses’ beliefs about the consequences of mobilization.

From the barriers and facilitators interviews with nurses on the unit, the KT intervention development team also noted that the nurses’ intersecting categories of age and education level were particularly important. Younger nurses were more likely to believe that ambulating older adults would result in moe falls while nurses with graduate degrees were less likely to hold this belief.

Table 3 provides the MOVE case example of how barriers, facilitators, and intersectionality considerations were summarized for the Moe study and includes a blank column for users to fill in.

**Table 3:** Summarizing barriers, facilitators, and intersectionality considerations for your knowledge translation (KT) project [26]

**Questions to ask**	**Mobilization of Vulnerable Elders (MOVE) case example**	**Your KT Project**
What barriers to behaviour change did you identity? These can be identified through knowledge syntheses, conversations with stakeholders, interviews/focus groups, surveys, and observations.	Belief that mobilizing patients will lead to more falls.	
Who is changing their behaviour?^a^	Unit 2A nurses	
What does an intersectional approach tell us about these barriers? Think through how you can identify barriers and their related context.	The education system (e.g., undergraduate nursing education) and organizational context (e.g., falls prevention policies at the hospital) support the belief that mobilizing patients will lead to patients falling. Middle-aged female nurses, who have historically held roles as caregivers to aging relatives, share stories of how mobilizing family members has led to falls.	
What facilitators to knowledge use did you identify?	Nurses’ desire to improve patient outcomes. Nurses’ desire to be in compliance with hospital’s falls prevention policies.	
What does an intersectional approach tell us about these facilitators?	Nurses’ motivation to provide quality care is driven by the intersection of their professional role, individual values, and societal norms. Nurses’ role as paid employees of the organization impacts their desire to comply with existing organizational mandates (e.g., falls prevention initiatives).	

^a^There can be many “whos” (e.g., nurses, doctors, administrators, people with lived experiences). Complete a table for each group that will be making a behaviour change

Table 4 outlines the process of identifying “what” and “who” will be targeted when designing a KT intervention with an intersectionality lens along with a blank column for users to answer the questions outlined in the first column.

**Table 4:** Clarifying the “what” and “who” for your knowledge translation (KT) project with an intersectional lens [26]

**Questions to ask**	**Mobilization of Vulnerable Elders (MOVE) case example**	**Your KT Project**
What is the current practice?	Patients’ mobility is not assessed upon admission or within 24 hours of admission. Mobility may be assessed at a later point for specific clinical cases.	
What is the behaviour you want to change?	Assessing mobility within 24 hours of a patient’s admission.	
How will the current practice be changed: • Stopped • Replaced • Modified • Added to	The current practice will be modified.	
Who is changing their behaviour?^a^	Nurses	
What are key intersecting categories as identified by those expected to change their behaviour? Note that the number of intersecting categories to consider will depend on the project. For more information on exploring intersecting categories, please visit the Intersectionality Guide [26].		

^a^There can be many “whos” (e.g., nurses, doctors, administrators, patients). Complete a table for each group that will be making a behaviour change

The MOVE team then went on to select and tailor a KT training and education intervention accounting for the barriers, facilitators, and intersectionality considerations identified. A full description of this process can be found in the Selecting and Tailoring KT Interventions Workbook developed for our larger Intersectionality & KT project [41].

## Discussion

This article describes the process used by, and deliberations of, our group to develop a tool for using an intersectionality lens with the TDF in order to enhance the science and practice of KT. The expert consensus process identified specific guiding questions for each theoretical domain. Our intent was for researchers, practitioners, and policy-makers engaging in KT to be able to use the tool presented here as a guide for incorporating intersectionality into their own work.

Based on its content and widespread use [5, 42, 43], the TDF is a useful exemplar for enhancing a KT framework with intersectional considerations. While at first consideration the TDF may seem ‘individual-focused’, many TDF domains lend themselves to greater considerations of broader intersecting social factors, if operationalized accordingly. Specifically, the TDF domain of Social Influences has a number of constructs that speak to social factors, including ‘Power/Hierarchy’, ‘Group conformity’, ‘Social Pressure’, ‘Social norms’, ‘Social Pressure’. Similarly, the Social/Professional Role/Identity domain includes the following constructs: ‘Identity’, ‘Alienation’, ‘Group identity’. Intersectionality provides a way to further explore elements of context relevant to the barriers assessment phase of implementation across micro, meso and macro levels. The considerations suggested within our tool draw attention to intersecting social factors and may encourage users to reflect on their implications on a wider scale. In addition, our tool can encourage users to think about interactions between domains (e.g. between “identity” factors and social influences”) and how power structures may play a role in these interactions.

While individuals’ demographic characteristics have previously been classified as one element of context [6], there has been limited discussion regarding the broader social implications of these demographics and especially their intersections. Intersectionality is more than just identifying independent sociodemographic factors – it is about the synergy of these factors within the individual as they relate to broader societal system. Further, when focusing on the behaviour of individuals, there is a risk that the behaviour can become “de-contextualized” from larger social structures [44]. In other words, the individual patient or clinician may be placed at the centre of the “problem” without exploring the larger context influencing barriers to the target behaviour. Larger systems and structures of power shape the social context in which interventions are implemented (e.g., ageism, sexism, ethnocentrism). For example, a racialized, immigrant home care worker may not be able to freely participate in surveys, interviews and other KT activities implemented by Canadian administrators for fear of affecting employment or citizenship status. Applying an intersectionality lens to the TDF enables factors such as these to be considered more explicitly in assessments of barriers and enablers and subsequent design of implementation interventions. In addition, the prompts proposed in our enhanced version of the TDF may help to improve the quality of the information gained when conducting TDF surveys or interviews.

Our intent is not to replace the original TDF, but rather, to augment it by providing researchers and intervention developers complementary options, particularly when the problem they are targeting may be affected by intersecting categories and social structures. Our tool can also encourage users to think about intersectionality as it relates to a particular problem or intervention when they may not have previously considered it. Importantly, our intersectionality tool does not change the fundamental components of the TDF, but instead helps users consider the ways in which these existing barriers and facilitators are experienced and described in each TDF domain may play out differently based on people’s individual experiences of intersectionality. The approach suggested here may be used as a starting point as others may wish to modify or consider intersectional considerations differently when working with the TDF or additional MTFs. Importantly, a number of potential prompts are provided in order to catalyze reflection by users regarding an array of potential intersectional factors that could influence their KT project. Given that each project is unique, users are encouraged to read and reflect on all prompts but select those which are a pertinent ‘fit’ with the context(s) of their specific project. There may be specific social positions or identities that are particularly relevant to the research question and our tool can provide options to users for exploring those particular aspects.

### Limitations and strengths

We recognize that it may seem cumbersome to consider all 53 prompts alongside standard operationalizations of TDF topic. The selection of the prompts to use rests with the intervention developers based on their implementation context. We do not expect all 53 prompts to be used in any single given study. While there might be some repetition among the prompts, participants felt it was important to take a comprehensive approach to ensure these aspects were considered where they might be relevant. It will be up to the research teams who use this tool to subsequently decide which domains they may need to consider in a particular project. Repetition of some prompts across domains can help to ensure key considerations are not missed. We also recognize that using intersectionality and the TDF can be challenging, and additional training or support may be required to facilitate successful use of these approaches in practice.

Although our approach is limited by the relatively small number of experts involved in the consensus process, those involved represent a diverse range of academic disciplines, experiences and intersectional categories. In the future, researchers may wish to further refine the work presented here through a larger consensus process with even greater diversity. It will also be important to study user perspectives and experiences when using operationalizing the TDF with and without intersectionality-prompts. The next phase of this project is to conduct pilot testing with KT practitioners developing and implementing KT interventions. Future research could compare the original TDF and the intersectionality-enhanced TDF to determine whether any new information gained would affect the nature of the intervention developed and its ultimate impact. Studies could compare differences in the results of interviews focusing on the same topic when using the intersectionality prompts versus the original TDF. Comparisons of intervention development following each approach could also be conducted. Researchers may also wish to determine whether specific interventions are needed to address multiple domains impacted by overlapping social and power-related factors.

When using intersectionality to enhance KT MTFs, researchers may wish to consider the implications of engaging MTF originators, particularly if they decline support. In our case, the TDF originator was supportive of the work. A larger question for the KT community to address how we can continue to advance the science and practice of KT with intersectionality even when it modifies the original intended use of prior work. From our perspective, using an intersectionality lens alongside the TDF is a step toward a more contextualized and inclusive KT. If the main purpose of theory is to be a representative summary of factors known to affect a given phenomenon [45], it stands to reason that incorporating factors that tend to be under-represented to date using intersectionality can serve to continue to refine theory.

Finally, it will be important for researchers to continue to work with many diverse groups to understand how to use intersectionality alongside the TDF while respecting nation or culture-specific knowledge systems. Please see Appendix for a project limitations statement as it relates to our context in Canada.

## Conclusions

Through an expert-consensus approach, we developed a tool for applying an intersectionality lens to the TDF. Considering the role of intersecting social factors when identifying barriers and facilitators to implementing research evidence may result in more targeted and effective interventions that better reflect the realities of those involved.

## Data Availability

The datasets used and/or analysed during the current study available from the corresponding author on reasonable request.

## Appendix

### Project Limitations Statement

We acknowledge that the work of our Canadian Institutes of Health (CIHR)-funded team grant was conducted on unceded lands that were the traditional territories of many people, including the Algonquin, Cree, Dakota, Dene, Huron-Wendat, Mississaugas of the Credit River, and the Musqueam Peoples, and on the homeland of the Métis Nation. We acknowledge the harms of the past and the harms that are ongoing. We are grateful for the generous opportunities to conduct work on these lands.

In 2017, the CIHR launched an opportunity for team grants in gender and KT. This opportunity (sponsored by the Institute of Gender and Health) was developed to recognize that the field of KT had yet to thoughtfully integrate gender into its research agenda. The objectives of the CIHR team grant competition were to generate evidence about whether applying sex- and gender-based analysis to KT interventions involving human participants improves effectiveness, thereby contributing to improved health outcomes; contribute to a broader knowledge base on how to effectively and appropriately integrate gender into KT interventions; and facilitate the consideration and development of gender-transformative approaches in KT interventions.

In response to this call, we submitted a grant aimed at helping KT intervention developers use an intersectional approach when designing and implementing interventions to address the needs of older adults. We received feedback from the CIHR peer review committee that substantial concern was raised about our focus on intersectionality. In particular, the Scientific Officer’s notes described that the focus on intersectionality would dilute the focus on gender and needed to be reconsidered. A meeting was subsequently held with the successfully funded team and this issue was raised again. We acknowledge the limitation that our intersectional approach comes at the expense of a minimized focus on gender. However, because intersecting categories, such as gender and age, are experienced together, we ultimately elected to use an intersectional approach as it encapsulates the lived experience of those we aim to impact.

A more significant limitation of our work is that we did not include First Nations, Inuit, and Métis community members in the grant proposal. As such, their needs and perspectives were not included in the research grant and, consequently, funded activities. Our team did not have established relationships or expertise in this area and as such, we felt it was inappropriate for our team to work on a grant in this area.

We strongly believe that consideration of gender and KT for Indigenous peoples should be a primary focus of a distinct team grant.

There are established best practices for community engagement with First Nations, Inuit, and Métis Peoples that begin with principles of collaboration, which take time to develop and must not be tokenistic. The principles for collaboration should ensure authentic engagement, shared respect, trust, and commitment to ensure long-term, mutually empowered relationships. These principles should also ensure that the research-related priorities meet the needs, perspectives, and expectations of the First Nations, Inuit, and Métis Peoples. Indigenous peoples have a long history of conducting research, and this tradition continues today with many Indigenous healers and scholars leading research in various areas. Indeed, there are many Indigenous scholars working in the KT field.

Because the team’s work did not include First Nations, Inuit, and Métis Peoples and involve adhering to the principles that guide their engagement in research, the needs and considerations of these Peoples were not included in the work conducted in this team grant. As such, anyone who is considering using the outputs of this team grant needs to know that they cannot be broadly applied to these Peoples and there may be other more culturally appropriate models/theories/frameworks that are useful to consider. Similarly, because this research focused on older adults (and in particular, chronic disease management in older adults) it does not apply to children and youth.

We believe that any KT intervention work needs to begin with engaging the appropriate community and is only applicable when those communities are engaged throughout the research enterprise. Moreover, intersectionality involves deep immersion in the lived experiences and priorities of those communities. As a result, KT work requires immersive work with various populations and not just key informants to ensure the work meets the needs of the relevant populations.

We thank and acknowledge Dr. Lisa Richardson, Co-Lead, Indigenous Health Education, Faculty of Medicine, University of Toronto, for her time and expertise in reviewing this statement.

## Abbreviations

BCW: Behavior Change Wheel
KT: knowledge translation
MTFs: models, theories, frameworks
TDF: Theoretical Domains Framework
